# Congenital multiple colonic atresias with intestinal malrotation: a case report

**DOI:** 10.1186/s40792-020-00822-z

**Published:** 2020-03-30

**Authors:** Daisuke Ishii, Hisayuki Miyagi, Masatoshi Hirasawa, Kazutoshi Miyamoto

**Affiliations:** Department of Pediatric Surgery, Asahikawa Medical University, 2-1-1, Midorigaoka-higashi Asahikawashi, Hokkaido, 078-8510 Japan

**Keywords:** Colonic atresia, Multiple, Intestinal malrotation, Microcolon

## Abstract

**Background:**

Congenital intestinal atresia develops in 1 in 1500 to 20,000 births. Colonic atresia, which accounts for 1.8–15% of intestinal atresia cases, is accompanied by other gastrointestinal atresias such as small intestinal atresia, gastroschisis, imperforate anus, and intestinal malformation in 47–80%. Although a report shows that patients with multiple colonic atresias are 8.9% of those with colonic atresia.

**Case presentation:**

A male infant did not have the first bowel movement within 36 h of birth and had abdominal distention/vomiting. Radiography showed significant dilation of the intestinal tract. A contrast enema examination at 3 days of age showed a microcolon and disruption in the descending colon. We performed an emergency decompressive loop enterostomy in the distended segment. At the age of 7 months, imaging from the stoma showed disruption of the contrast medium in the intestinal tract at the right lower abdomen, and the continuity of the intestinal tract was not clarified. Intestinal malrotation was found during the second surgery, and the enterostomy was located in the ileum proximal to Bauhin’s valve. Continuity of the intestinal serosal surface was maintained. However, multiple membranous obstructions (three atresias and one stenosis) were observed in the distal segment of the bowel, which was penetrated by intraluminal advancement of a urethral catheter. Therefore, he was diagnosed with multiple colonic atresias. The intestinal tract was longitudinally incised, and membranectomy and mucosal/lateral suture were performed.

**Conclusions:**

It is important for neonates with intestinal atresia to evaluate and prepare for distal patency of the colon before radical anastomosis. In addition, anomalies associated with colon atresia should also be assessed.

## Background

Congenital intestinal atresia develops in 1 in 1500 to 20,000 births [1, 2]. Colonic atresia, which accounts for 1.8–15% of intestinal atresia cases [3, 4], is accompanied by other gastrointestinal atresias such as small intestinal atresia, gastroschisis, imperforate anus, and intestinal malformation in 47–80% [5]. Although a report shows that patients with multiple colonic atresias are 8.9% of those with colonic atresia [6], there have been three reports of multiple colonic atresias with malrotation according to our most extensive search. However all three reports were TypeIII atresias. We report a case of congenital multiple colonic atresias (TypeI) with intestinal malrotation, along with a literature review.

## Case presentation

**Fig. 1:**
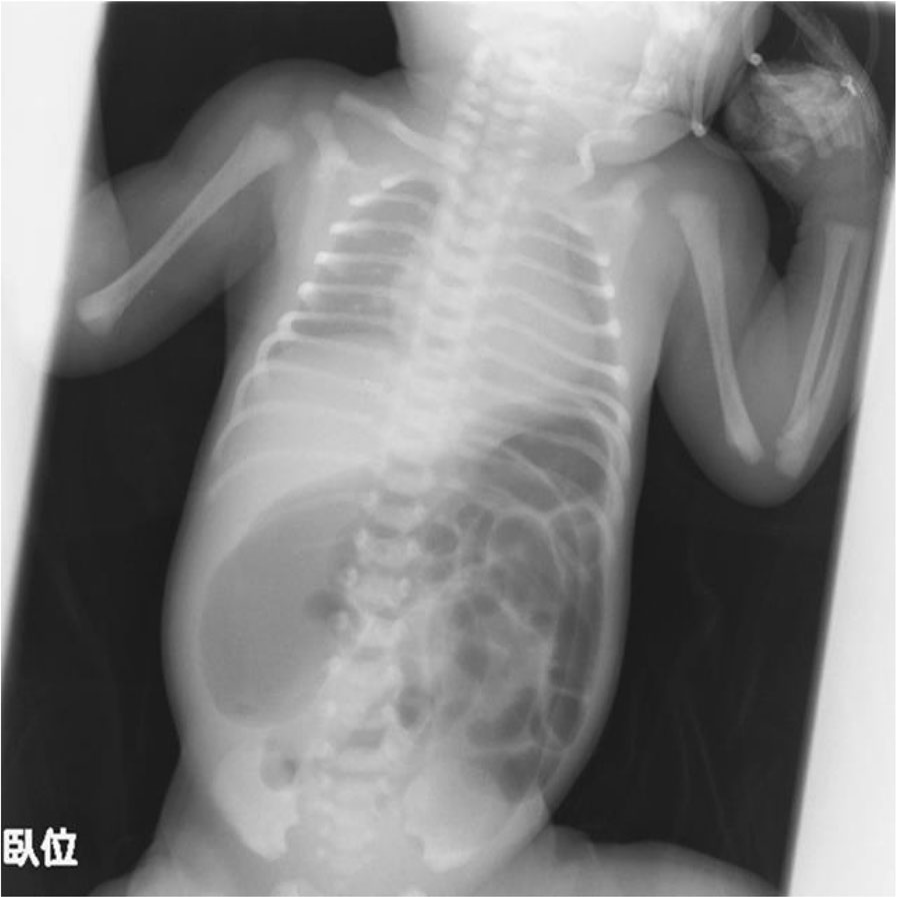
Plain abdominal radiography (36 h after birth). Plain abdominal radiography showed a significant dilation of the intestinal tract and no gas in the pelvic cavity

**Fig. 2:**
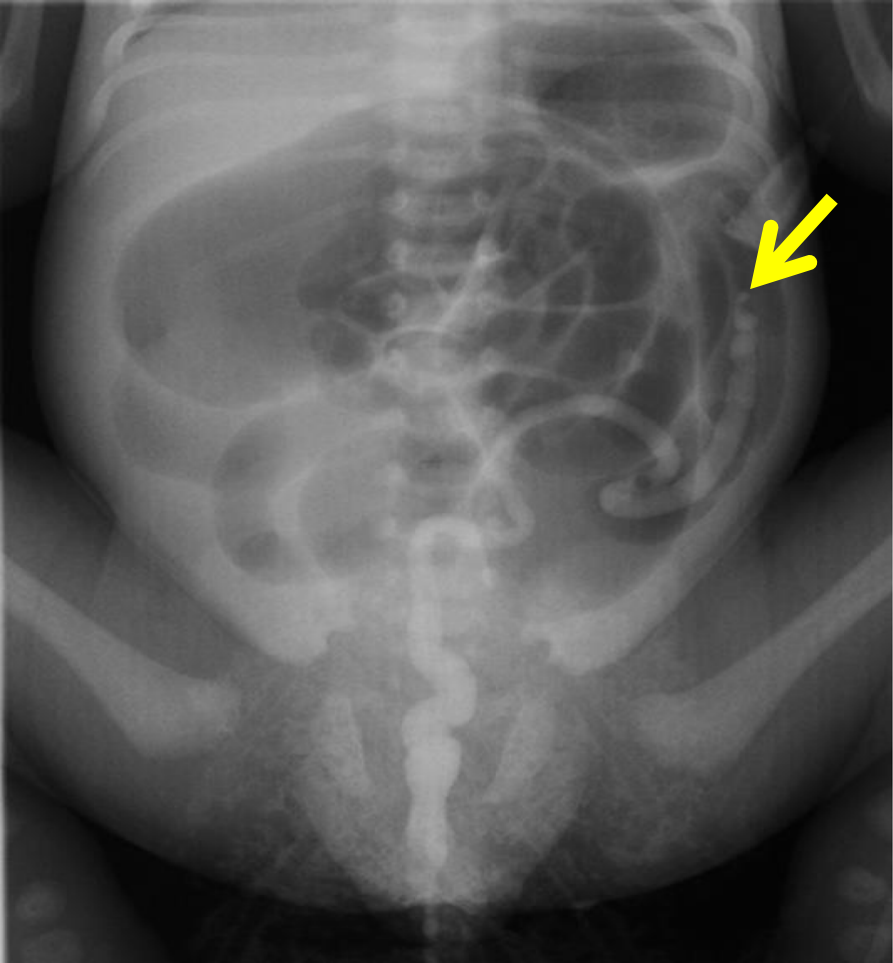
Contrast enema examination (3 days of age). The arrow showed a microcolon and disruption in the descending colon

The course of pregnancy was normal. The infant was born at a gestational age of 38 weeks and 6 days by normal vaginal delivery, and birth weight was 2790 g at the previous hospital. The infant did not have the first bowel movement within 36 h of birth and had abdominal distention and vomiting. Plain abdominal radiography showed significant dilation of the intestinal tract and no gas in the pelvic cavity (Fig. 1), which did not respond to catheterization with a Nelaton’s catheter or enema. A contrast enema examination at 3 days of age (Fig. 2) showed a microcolon and disruption in the descending colon. Taking intestinal atresia, Hirschsprung’s disease, and hypoplastic left hemicolon as a preoperative diagnosis into consideration, he was transferred to our hospital.

Because of his unsteady breathing condition, we performed emergency enterostomy at 3 days after birth. Laparotomy, with 3 cm transection in the upper right abdomen, observed serous ascites, but no adhesion was observed. After puncturing the dilated intestinal tract and depressurizing, we performed only emergency enterostomy with double orifices for the dilated intestinal tract. During the operation, we wanted to confirm distal patency and associated anomalies and make a definitive diagnosis, but we could not do it due to prominent intestinal dilatation and his unsteady breathing condition.

A biopsy of the intestinal tract at the site of enterostomy showed ganglion cells in the Meissner’s and Auerbach’s plexus. And a biopsy of the rectal mucosa showed ganglion cells in the Meissner’s plexus. Therefore, Hirschsprung’s disease was ruled out. As associated anomalies, the infant had Ebstein’s anomaly (mild) and mild pyelectasis on the left side (I–II). His family history was noncontributory.

**Fig. 3:**
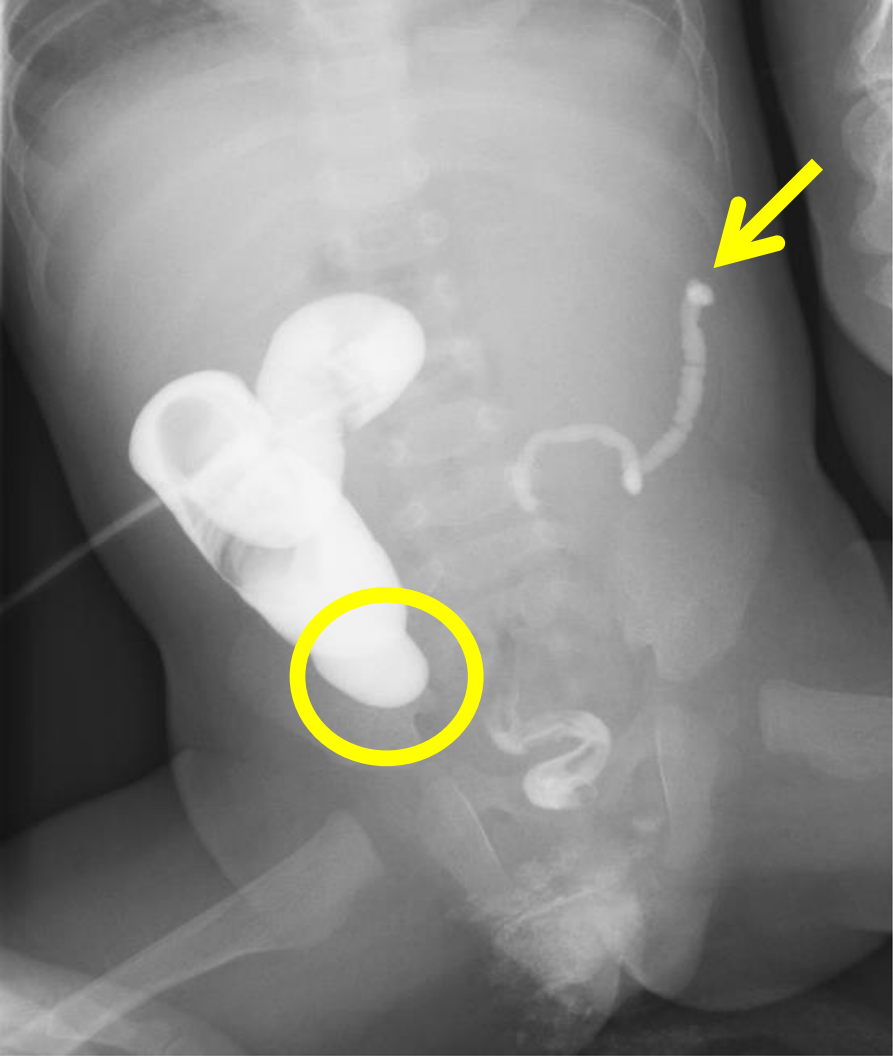
Preoperative contrast enema examination (7 months of age). Imaging from the anus showed a microcolon and disruption of the contrast medium in the descending colon (arrow). Imaging from the stoma showed a disruption of the contrast medium inside the intestinal tract in the right lower abdomen (circle), and the continuity of the cecum and intestinal tract on both sides was not clear

He was admitted to our department to undergo radical operation at 7 months of age. The height was 65.1 cm and weight was 6.53 kg. Preoperative contrast enema from the anus showed a microcolon and disruption of the contrast medium in the descending colon. Imaging from the stoma showed a disruption of the contrast medium in the intestinal tract at the right lower abdomen, and the continuity of the intestinal tract on both sides was not clear (Fig. 3).

**Fig. 4:**
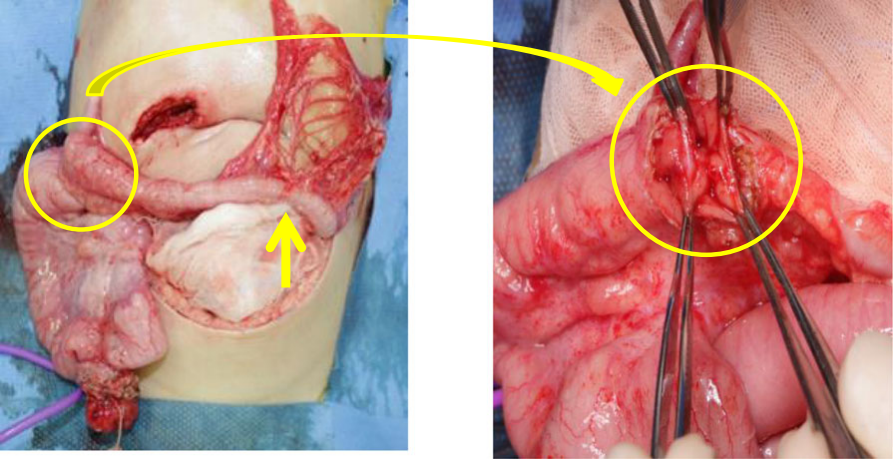
Operative findings. The continuity of the intestinal serosal surface was maintained, while, as for the lumen, there were membranous atresias in the Bauhin’s valve and anus, 2 cm from the Bauhin’s valve (circle), and a membranous atresia in the transverse colon and stricture in the anus, 2 cm from the atresia (arrow)

Laparotomy, with transection in the lower abdomen, showed a complicated non-rotation type of intestinal malrotation, and the enterostomy was located in the ileum proximal to Bauhin’s valve. We removed the ileostomy once to confirm distal patency. The continuity of the intestinal serosal surface was maintained, whereas in the lumen, there were 3 membranous atretic sites and 1 stenotic site; (1) a membrane-like atresia in the Bauhin’s valve, (2) a membrane-like atresia in the 2-cm anal side of Bauhin’s valve, (3) a membrane-like atresia in the transverse colon, and (4) a stricture in the 2-cm anal side of the atresia (Fig. 4). For the three membrane-like atresias (TypeI), the intestinal tract was longitudinally incised, and membranectomy and mucosal/lateral suture were performed. We only performed longitudinal incision/lateral suture for the constricted intestinal tract. Finally, the ileostomy was reconstructed at the same ileal site. The operative time was 2 h 59 min, and blood loss was 55 g. The postoperative course was favorable, and he was discharged on day 14 postoperatively.

**Fig. 5:**
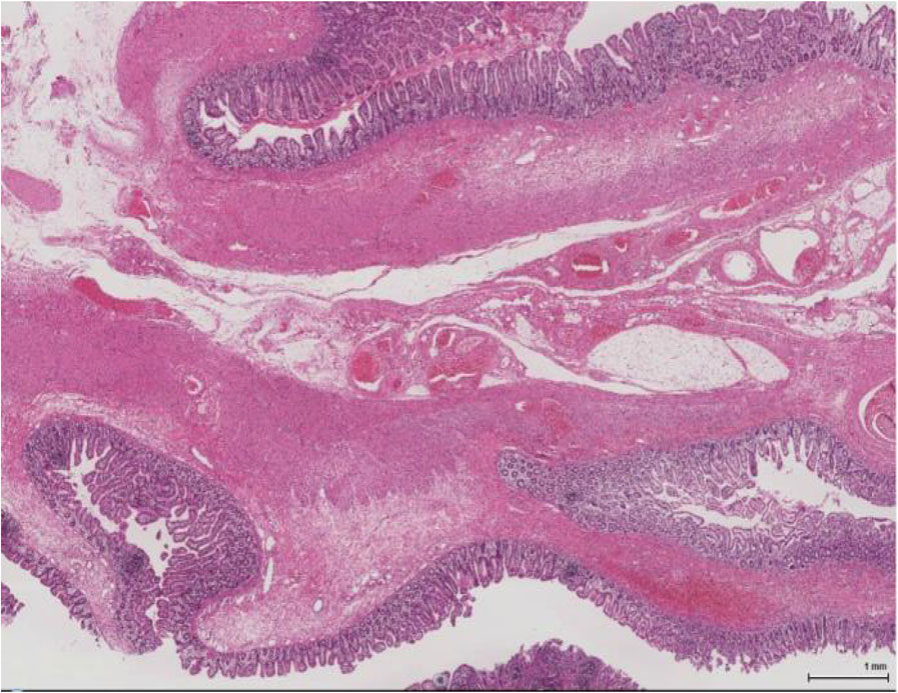
Pathological diagnosis. The wall structure in membranous atresias was maintained, and mucosal epithelial regenerative changes were observed. In the resected membranes of the atresias in the Bauhin’s valve and ascending colon, the muscle layer structure was unclear in the entire specimens, and the muscle layer was occupied with hyperplastic connective tissue

Pathologically, the wall structure of the membrane-like atresias was maintained, and mucosal epithelial regenerative changes were observed (Fig. 5). Ganglion cells were present in the Meissner’s and Auerbach’s plexus. In the resected membranes of the atresias in the Bauhin’s valve and ascending colon, the muscle layer structure was unclear in the entire specimens, and the muscle layer was occupied by hyperplastic connective tissue, mainly composed of collagen fibers, in which defect and developmental abnormalities of the muscle layer were suspected as the causes of atresia.

At 9 months of age, he was admitted for the ileostomy closure. Simulated stool had been injected into the intestinal tract in the anal side of the stoma 7 days before surgery. A contrast enema examination confirmed the improvement in disuse atrophy of the intestinal tract and its passage. But at operation a stricture was observed in the region where the membrane-like atresia in the transverse colon was removed. We performed a balloon dilation and the ileostomy closure. The postoperative course was favorable, and the baby was discharged on day 11 after surgery. Currently, he has outpatient follow-up visits, and the control of defecation is favorable.

## Discussion

Colonic atresia, a type of congenital intestinal atresia that develops in the colon, was first reported by Binninger et al. in 1873, and a lifesaving case was reported by Gaub et al. in 1922 [7]. Congenital intestinal atresia develops in 1 in 1500 to 20,000 births [1, 2], and colonic atresia is shown to account for 1.8–15% of them [3, 4]. We had 75 patients with congenital intestinal atresia between 1989 and 2017 in our department, of whom, five patients (6.7%) had colonic atresia. Although a report shows that patients with multiple colonic atresias are 8.9% of those with colonic atresia [6], there have been three reports of multiple colonic atresias with malrotation according to our most extensive search (Table 1) [8, 9] (excluding patients with familial intestinal polyatresia (FIPA) syndrome stated below). All three reports were TypeIII atresias with our case being the first reported case of TypeI atresias.

**Table 1:** Reports of multiple colonic atresias with malrotation

	Birth weight	Gastational age	Time of first surgery	Atresias	Type	First surgery	Anomalies	Ref
1	nm	40 weeks	52 h	A, T, D	III	Ostomy	Malrotation	8
2	nm	40 weeks	48 h	T, Sp	III	Ostomy	Malrotation	8
3	nm	34 weeks	12 h	A, H, T	III	Ostomy	Gastroschisis, malrotation	9
4	2790 g	38 weeks	72 h	A, T	I	Ostomy	Ebstein’s anomaly, pyelectasis, malrotation	Our case

*nm* not mentioned, *A* ascending colon, *T* transverse colon, *Sp* sigmoid colon, *D* descending colon

The causes of congenital intestinal atresia are explained by the theory of developmental anomaly due to failure of recanalization by Tandler et al. [10] and the theory of vascular insufficiency by Louw et al. [11]. In the present case, atresia was categorized as type I in Louw’s classification; there was no finding that suggests vascular insufficiency, including no abnormality in the mesentery; and the pathological diagnosis showed findings in which developmental anomaly was suspected, so failure of recanalization was considered to be the cause of the disease. But we cannot explain the cause of multiple atresias because of only failure of recanalization. As a cause of multiple atresias, vascular insufficiency due to gastroschisis and FIPA syndrome [12, 13] are common causes to be discriminated. The present case had no findings to suggest them. So, multiple atresias can be caused by various factors. Further case accumulation and analysis are required.

Combined malformation is shown to occur in 47–80% of patients with colonic atresia [5, 6], and atresia of the other gastrointestinal tracts, such as small intestinal atresia, gastroschisis, imperforate anus, intestinal malrotation, cardiac malformation, omphalocele, and Hirschsprung’s disease, have been reported [6]. The most common of the combined malformation is gastroschisis. Intestinal malrotation is less common as the combined malformation [14, 15].

Since in colonic atresia, due to the presence of the Bauhin’s valve, which is different from intestinal atresia in other regions, closed loop obstruction occurs, the onset of abdominal distention and vomiting is delayed, and symptoms commonly occur after 24 h of birth. Therefore, necrosis and perforation easily occur, and early diagnosis is necessary. A report showed that, in patients who underwent surgery over 72 h after birth, the mortality is significantly higher than those who had surgery within 72 h after birth, and the cause of death is perforation due to dilation of the occluded colon in the rostral portion [8]. For the atresia in the right colon, one-stage surgery, in which anastomosis is performed at the first surgery, and for the atresia in the left colon, multiple-stage surgery, in which anastomosis is performed after colostomy, have been recommended [16]. However, one-stage surgery is advantageous in that it requires fewer surgeries, but the risks of ileocecal resection, anastomotic leak, and sepsis are shown to be high [6]. Multiple-stage surgery allows safe decompression of an anastomotic site as well as a dilated intestinal tract, and this is advantageous in the conservation of the ileocecal valve. However, it is disadvantageous in that it requires more number of surgeries. Because our patient had a significant dilation of the intestinal tract at the first surgery, we could not confirm distal patency and make a definitive diagnosis and we judged that the risk of complication would be high in one-stage surgery, and performed emergency enterostomy. In a case of mild dilation of the intestinal tract, one-stage surgery can be performed. However, in a case of potential multiple atresias, as in our patient, and a case of colonic atresia, combined malformation is common, and we consider that by performing multiple-stage surgery, radical operation, after a preoperative sufficient examination of the intestinal tract, is safe.

## Conclusions

It is important for neonates with intestinal atresia to evaluate and prepare for distal patency of the colon before radical anastomosis. In addition, anomalies associated with colon atresia should also be assessed. In a case of potential multiple atresias, we consider that by performing multiple-stage surgery, radical operation, after a preoperative sufficient examination of the intestinal tract, is safe.

## Data Availability

Not applicable.

## Abbreviations

FIPA: Familial intestinal polyatresia
